# Velocity neurons improve performance more than goal or position neurons do in a simulated closed-loop BCI arm-reaching task

**DOI:** 10.3389/fncom.2015.00084

**Published:** 2015-07-14

**Authors:** James Y. Liao, Robert F. Kirsch

**Affiliations:** ^1^Cleveland Functional Electrical Stimulation CenterCleveland, OH, USA; ^2^Department of Biomedical Engineering, Case Western Reserve UniversityCleveland, OH, USA

**Keywords:** neuron, brain computer interface, simulator, goal, velocity, position

## Abstract

Brain-Computer Interfaces (BCIs) that convert brain-recorded neural signals into intended movement commands could eventually be combined with Functional Electrical Stimulation to allow individuals with Spinal Cord Injury to regain effective and intuitive control of their paralyzed limbs. To accelerate the development of such an approach, we developed a model of closed-loop BCI control of arm movements that (1) generates realistic arm movements (based on experimentally measured, visually-guided movements with real-time error correction), (2) simulates cortical neurons with firing properties consistent with literature reports, and (3) decodes intended movements from the noisy neural ensemble. With this model we explored (1) the relative utility of neurons tuned for different movement parameters (position, velocity, and goal) and (2) the utility of recording from larger numbers of neurons—critical issues for technology development and for determining appropriate brain areas for recording. We simulated arm movements that could be practically restored to individuals with severe paralysis, i.e., movements from an armrest to a volume in front of the person. Performance was evaluated by calculating the smallest movement endpoint target radius within which the decoded cursor position could dwell for 1 s. Our results show that goal, position, and velocity neurons all contribute to improve performance. However, velocity neurons enabled smaller targets to be reached in shorter amounts of time than goal or position neurons. Increasing the number of neurons also improved performance, although performance saturated at 30–50 neurons for most neuron types. Overall, our work presents a closed-loop BCI simulator that models error corrections and the firing properties of various movement-related neurons that can be easily modified to incorporate different neural properties. We anticipate that this kind of tool will be important for development of future BCIs.

## Introduction

Brain-Computer Interfaces (BCI) are systems that record electrical signals from the brain and relate information in these signals to intended actions such as arm movements (Bansal et al., 2012; Hochberg et al., 2012; Collinger et al., 2013; Nakanishi et al., 2013) or communication (Santhanam et al., 2006; Krusienski and Wolpaw, 2009). Two landmark studies have shown that BCIs can provide a means for people with tetraplegia to command prostheses like robotic arms (Hochberg et al., 2012; Collinger et al., 2013) and could allow these individuals to perform functional tasks of daily living by controlling such assistive devices. BCIs have large potential benefits for individuals with tetraplegia, particularly those with high cervical level injuries or brainstem stroke, because these individuals have relatively few muscles or movements under volitional control that could otherwise be used to command a prosthetic device.

Many studies, including Collinger et al. (2013) and Hochberg et al. (2012), assume that firing rates of individual neurons recorded from primary motor cortex are linearly related to the kinematics of the robot hand. Specifically, they assume that the neurons are directionally cosine-tuned and gain-modulated by the magnitude of the kinematic variable (Kettner et al., 1988; Schwartz et al., 1988; Moran and Schwartz, 1999; Wang et al., 2007).

One potential way to improve the performance of these BCI systems is to utilize qualitatively different kinds of information. The brain contains many movement-related signals in addition to the well-characterized continuous movement kinematics on which existing studies have relied. In the context of restoring arm reaching movements, signals that represent the movement goal are particularly relevant because for such movements the target is usually more functionally relevant than the specific trajectory used to reach the target. Obtaining accurate information related to movement goal would greatly simplify the user interface, as the participant would simply think about the intended goal and the assistive device generating the movement [e.g., a functional electrical stimulation system (Memberg et al., 2014) or a robotic exoskeleton] would automatically generate the needed trajectory and use feedback control to guide the arm to this goal location with minimal ongoing input from the user. A variety of brain areas contain information about the movement goal (Alexander and Crutcher, 1990; Shen and Alexander, 1997; Hatsopoulos et al., 2004; Saito et al., 2005; Pesaran et al., 2006; Santhanam et al., 2006; Yu et al., 2007; Mulliken et al., 2008a,b; Bhattacharyya et al., 2009; Shanechi et al., 2013a) and there are several ways to refine the decoded hand trajectory using the available goal information (Hatsopoulos et al., 2004; Srinivasan and Brown, 2007; Yu et al., 2007; Kulkarni and Paninski, 2008; Corbett et al., 2012; Lawhern et al., 2012; Shanechi et al., 2013b). In particular, a recent study showed that human parietal cortex contains goal-tuned neurons that can perform a closed-loop goal selection task (Aflalo et al., 2015).

Although it is clear that goal-related signals can be recorded from the brain, it is currently unclear how useful goal-tuned neurons would be, relative to position or velocity-tuned neurons, as inputs to a BCI for controlling arm movements. Exploring the likely effectiveness of goal-tuned neurons is thus important for justifying the future targeting of recording electrode implants to brain areas that contain goal-tuned cells. The purpose of the current study was to provide some insight into this issue by developing a simulation of a closed-loop BCI for controlling arm movements. This simulation included a submovement-based movement controller (Liao and Kirsch, 2014) that was trained on experimental movements, an encoder of neural firing rate modulation and noise properties based on literature reports, and an Extended Kalman Filter decoder that had the same structure as the encoded neural properties (i.e., it was assumed that the ideal decoding structure was known *a priori*). We then used this simulator to predict the relative contributions of various combinations of neurons modulated by position, velocity, and movement goal to overall BCI performance, as well as the impact of the number of neurons of each type on performance.

## Materials and methods

**Figure 1:**
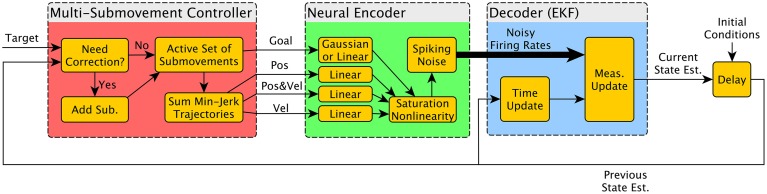
**Diagram of simulator components**. The BCI simulator contains three major components. The Multiple Submovement Controller (red) generates realistic trajectories from a starting position to a target position, and the resulting position, velocity, and goal trajectories are used as inputs by the Neural Encoder (green) to drive the firing rates of neurons that are Gaussian or Linear tuned. These nominal firing rates are converted into noisy firing rates and finally decoded by an Extended Kalman Filter (EKF). The decoded state vector (labeled “Current State Est.”) contains Position, Velocity, Acceleration, and Goal, and is fed back to the Multiple Submovement Controller to generate possible error corrections. It is also fed back to the EKF so that the estimate can be iteratively improved at each timestep. The delay box at the right completes the diagram and is how the state estimate at the current timestep is passed into the simulation at the subsequent timestep.

**Figure 2:**
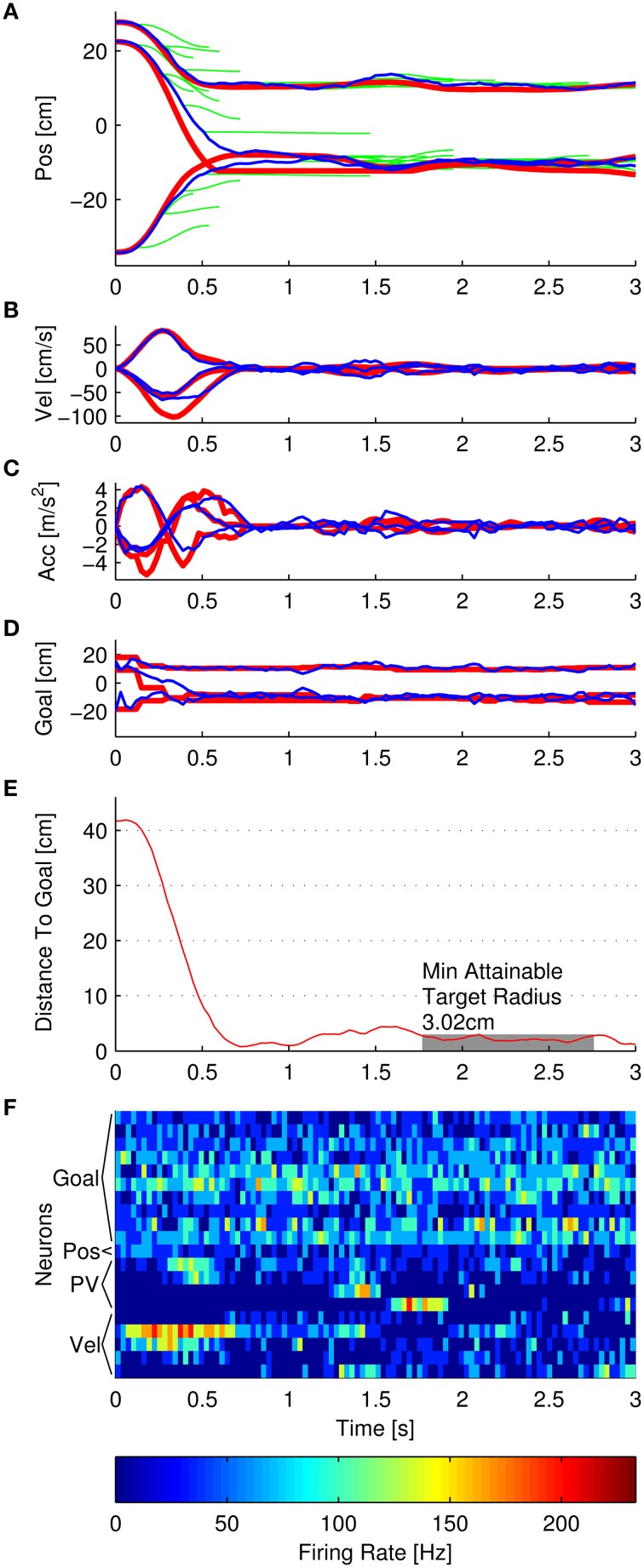
**Example of a single simulated reach**. **(A–F)** represent position, velocity, acceleration, goal, distance to goal, and noisy firing rates, respectively. In each panel, the red traces represent the trajectory that the Multiple Submovement Controller commanded. **(A)** also contains green traces that represent the individual submovements that compose the commanded trajectories. The blue traces represent the respective trajectories decoded by the Extended Kalman Filter. The distance to goal panel also indicates that the minimum attainable target radius for this particular simulation was 3.02 cm, and the initial distance to goal was 41.76 cm. **(F)** indicates the noisy firing rates corresponding to 10 goal neurons (Gaussian tuned with 20 cm standard deviations), 1 position-only neuron, 4 position and velocity neurons (labeled PV), and 5 velocity-only neurons.

A schematic of the simulator is shown in Figure 1. The three main components were the Multiple Submovement Controller (MSC), the Neural Encoder, and the Decoder. The MSC (Liao and Kirsch, 2014) is a submovement-based model of error corrections during human movements that provided movement commands in the form of position, velocity, and goal, as a function of the target position, start position, and the decoded position, velocity, acceleration, and goals that were fed back to the MSC from the Decoder. The Neural Encoder generated firing rates based on the movement commands computed by the MSC, the modulation properties of cortical neurons as extracted from previous studies, and spiking noise (see below). Finally, an Extended Kalman Filter (the Decoder) decoded the noisy firing rates into the aforementioned position, velocity, acceleration, and goal signals. One complete simulated reaching movement is represented in Figure 2, including the three dimensional kinematics *commanded* by the MSC (Figures 2A–D, red traces), the firing rates generated by the Neural Encoder (Figure 2F), and the *decoded* kinematics (Figures 2A–D, blue traces). The simulator components and their representations on Figures 1, 2 are discussed in more detail below. Sample code is available in the Supplementary Materials.

### Multiple submovement controller

The MSC (Liao and Kirsch, 2014) generates realistic reaching trajectories and consists of three Artificial Neural Networks (ANNs) that were pre-trained using experimentally recorded human reaching data to generate a command trajectory by linearly summing a discrete number of minimum-jerk submovements. This controller is based on a theory of human movement that represents movements as a set of overlapping-in-time submovements, each representing an error correction to the overall trajectory made in order to sustain progression to the target. Each submovement was evoked by kinematic features of the ongoing reaching movement. Specifically, one ANN predicted when a corrective submovement should be initiated and added to the current movement, and two additional ANNs predicted the durations and amplitudes of the new submovement, respectively. Each submovement was represented as follows:

(1) $$\begin{array}{l} pos_{i}\left(t\right)\text{​​​ ​}=d_{i}\left(6{\left(\frac{t-t_{0,i}}{t_{d,i}}\right)}^{5}\text{​​}-15{\left(\frac{t-t_{0,i}}{t_{d,i}}\right)}^{4}\text{​​}+10{\left(\frac{t-t_{0,i}}{t_{d,i}}\right)}^{3}\right),\\ \;t_{0,i}\leq t\leq \left(t_{0,i}+t_{d,i}\right)\\ pos_{i}\left(t\right)\text{ ​}=0,\;t<t_{0,i}\\ pos_{i}\left(t\right)\text{​ }=\text{​ }d_{i},\;t>\left(t_{0,i}+t_{d,i}\right) \end{array}$$

Here, the 3D position trajectory *pos*(*t*) of the *i* -th submovement was a minimum-jerk trajectory that started at time *t*_0,*i*_, had duration *t*_*d*,*i*_, and had 3D amplitude *d*_*i*_. Three separate ANNs predicted these parameters as functions of kinematic features of the movement. For instance, based on the start position, target position, decoded position, decoded velocity, decoded acceleration, and the predicted position at the end of the current submovements, an ANN predicted the next amplitude *d*_*i*_ to generate a submovement to bring the decoded position toward the target (see Supplementary Materials and (Liao and Kirsch, 2014) for a more detailed description).

Similar expressions to Equation (1) exist for the velocity and acceleration trajectories. The MSC generated a 3D command trajectory that was the linear summation of the individual submovements:

(2) $$\begin{array}{l} \;pos_{command}\left(t\right)=pos_{start}+\sum_{i}pos_{i}\left(t\right)\\ \;vel_{command}\left(t\right)=\sum_{i}vel_{i}\left(t\right)\\ \;acc_{command}\left(t\right)=\sum_{i}acc_{i}\left(t\right)\\ goal_{command}\left(t\right)=pos_{start}+\sum_{i}D_{i}\left(t\right)\\ \;D_{i}\left(t\right)=0,\;t<t_{0,i}\\ \;D_{i}\left(t\right)=d_{i},\;otherwise \end{array}$$

The commanded position trajectory was the sum of the individual submovement position trajectories *pos*_*i*_(*t*) initiated so far, plus the start position. The commanded velocity was the sum of the individual submovement velocities, and so on for acceleration. The commanded goal trajectory was the start position plus the sum of the amplitudes *D*_*i*_ of all the submovements that had initiated by time *t*. One additional restriction was that the commanded trajectories were limited to remain inside the workspace. The three red traces in Figures 2A–D represent the 3D commanded position, velocity, acceleration, and goal signals, respectively. In addition, the green traces in Figure 2A represent the individual MSC-generated minimum-jerk submovements that were summed to form the commanded position trajectory. For more examples of simulated trajectories, see Supplementary Figure S4.

### Neural encoder

The second component of the simulator was the Neural Encoder that simulated rate-based neurons that were tuned for position, velocity, or a combination of both position and velocity, in order to emulate known cortical cell populations. In addition, the Neural Encoder simulated neurons that were goal-only tuned. The encoding model generated firing rates for 30 ms timesteps. These nominal firing rates were then run through a saturation function to reflect physiologically feasible firing rates and then a spiking noise function to reflect the known (see below) noise properties of these neurons. Figure 2F shows an example of the noisy firing rates for simulated goal, position + velocity (labeled PV), position-only, and velocity-only neurons.

#### Position and velocity tuning

Position tuned cells were modeled as follows:

(3) $$f=f_{base}+f_{base}f_{depth}(x_{1}\sin q_{\theta }\cos q_{\phi }+x_{2}\sin q_{\theta }\sin q_{\phi }+\;x_{3}\cos q_{\theta })$$

**Figure 3:**
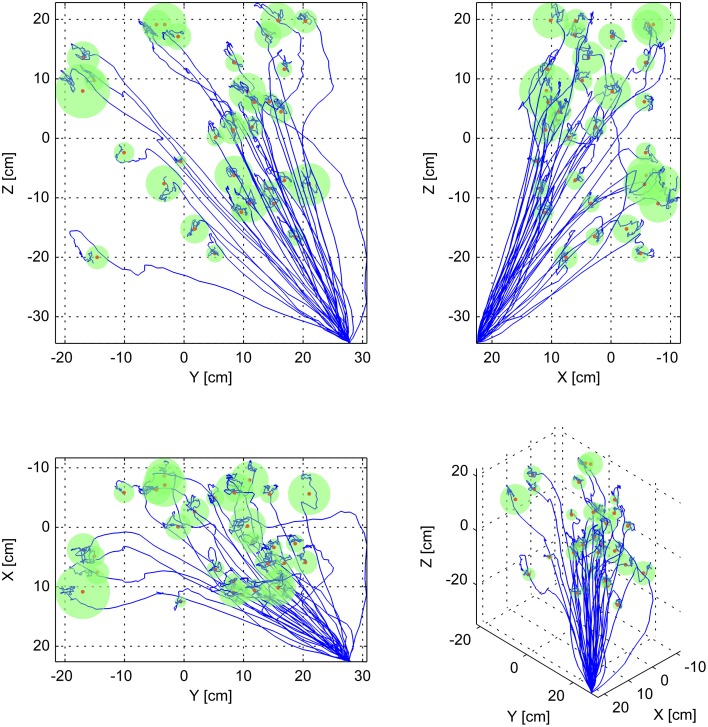
**Sample three-dimensional trajectories to all targets**. This figure shows all of the simulated decoded trajectories to all 33 targets, using one set of neuron parameters, and 3 position, 10 velocity, 7 position-velocity, and 20 goal neurons at 10 cm tuning width. Each subpanel represents a different view of the same data. The top-left panel represents viewing the data projected unto the Y-Z plane. The top-right panel represents the data projected unto the X-Z plane. The bottom left panel represents the data projected unto the X-Y plane. The bottom right panel represents a 3D view. In each panel the blue traces represent each of the decoded trajectories, and the green spheres with red centers represent each target. The radii of the spheres correspond to the Minimum Attainable Target Radius (MATR) for the corresponding simulation to each target. The mean MATR for this set of reaches is 2.22 cm. The workspace origin was located at (0, 0, 0).

Here, *q*_θ_ and *q*_φ_ represented the preferred direction of the neuron relative to the origin, *f*_*depth*_ represented the tuning depth, *f*_*base*_ represented the baseline firing rate, and *x*_1_, *x*_2_, and *x*_3_ represented the current position. This description, in spherical coordinates, is equivalent to gain-modulated cosine tuning (Moran and Schwartz, 1999). The origin (0, 0, 0) was set at the approximate center of the volume that contained the targets (Figure 3). For positions near the center of the workspace, the position neurons fired near their baseline rate. The firing rates increased for positions in the preferred direction from the origin, and decreased in the anti-preferred direction. For position tuning, *f*_*base*_ and *f*_*depth*_ were randomly sampled from exponential and gamma distributions previously reported (Wang et al., 2007), and the preferred directions were randomly sampled from the uniform spherical distribution.

Velocity-tuned neurons were also modeled using Equation (3), firing at baseline rates for zero velocity, increasing for velocities *x*_1_, *x*_2_, and *x*_3_ in the preferred velocity direction *q*_θ_ and *q*_φ_, and decreasing in the anti-preferred direction. As with position tuning, *f*_*base*_ and *f*_*depth*_ were randomly sampled from exponential and gamma distributions previously reported (Wang et al., 2007), and the preferred directions were randomly sampled from the uniform spherical distribution.

For the first analysis in this study (see below for description of the two analyses performed), we simulated a population of M1 neurons, which are known to contain a mixture of position-only tuned cells, velocity-only tuned cells, and cells that are tuned for both position and velocity (Wang et al., 2007). Based on the Wang study, our simulated M1 population was 50% velocity-only, 37% position and velocity, and 13% position-only tuned (note that these percentages exclude cells that Wang et al found had no velocity or position tuning). Cells tuned for both position and velocity were represented by:

(4) $$f=f_{pos}+f_{vel}-\frac{f_{base,pos}+f_{base,vel}}{2}$$

In this equation, *f*_*pos*_ and *f*_*vel*_ represent position and velocity-tuned components that were generated using Equation 3, and *f*_*base*,*pos*_ and *f*_*base*,*vel*_ represent corresponding baseline firing rates. The third term corrected the overall baseline to be the mean of the position and velocity baseline firing rates.

#### Goal tuning

In this study, we considered two kinds of goal tuning. First, we considered cortical neurons that were Gaussian tuned to specific locations in global space. These are representative of cells that have retinal receptive fields tuned for target position in an external reference frame (Galletti et al., 1993). As a simplification, this tuning model assumes that depth tuning is also Gaussian. There is also some evidence suggesting that M1 cells represent preferred spatial locations in this way (Aflalo and Graziano, 2006, 2007). Each Gaussian neuron was modeled as follows:

(5) $$f=f_{\text{min}}+f_{amp}\exp\left(\frac{{\left\|x-q_{pos}\right\|}^{2}}{-2q_{std}^{2}}\right)$$

Here, x represented the 3D intended goal, *q*_*pos*_ represented the 3D preferred position in space of this neuron (a randomly selected point in the 3D workspace), *f*_*min*_ was assumed to be 0 Hz, and *f*_*amp*_ was assumed to be 100 Hz. The tuning width was represented by *q*_*std*_ for which 10, 20, 30, and 40 cm were simulated.

Second, we considered a linear tuning function where the firing rate of each neuron was related to the direction and distance to the target. This is representative of the activity of goal-tuned cells in the dorsal premotor cortex (PMd) and is similar to the activity of gaze-related cells in several parietal areas (Sakata et al., 1980; Genovesio and Ferraina, 2004; Hadjidimitrakis et al., 2011) that contain neurons that are linearly or monotonically tuned for direction and depth, in three dimensions, of targets that are being fixated. Each linear goal neuron was modeled using Equation (3), where *q*_θ_ and *q*_φ_ represented the direction of the preferred goal from the origin, *f*_*depth*_ represented the tuning depth, *f*_*base*_ represented the baseline firing rate, and *x*_1_, *x*_2_, and *x*_3_ were the three coordinates of the intended goal. For intended goals near the center of the workspace, linear-tuned goal neurons fired near their baseline rate. The firing rates increased for intended goals in the preferred direction from the origin, and decreased in the anti-preferred direction. The linear tuning parameters *f*_*base*_ and *f*_*depth*_ were sampled from exponential and gamma distributions generated using the mean parameters reported for position and velocity neurons.

#### Saturation function and spiking noise

In order to limit the firing rates to physiologically realistic values, we applied an additional saturation function to the firing rates:

(6) $$\text{g}(x)=\frac{150}{1+9.305\exp{\left(-0.01602(x+190)\right)}^{6.015}}$$

These particular parameters of this generalized logistic curve were chosen to generate a sigmoidal saturation curve that converted large negative rates to zero, large positive rates to 150 Hz, and minimally affected moderate firing rates. For simplicity, we used the same saturation curve for all neurons. For the simulation, the spiking noise was either Poisson or Norm-Gaussian distributed according to Shoham et al. (2005)'s ratio.

### Decoding using Kalman Filter

The Decoder was an Extended Kalman Filter with a linear trajectory model and a nonlinear measurement model. The state vector included position, velocity, acceleration, and goal (Mulliken et al., 2008a; Corbett et al., 2012). The linear trajectory model was trained using least squares (Wu et al., 2006) using experimentally recorded trajectories augmented by a goal term derived from submovement decomposition (Liao and Kirsch, 2014), i.e., the same set used to train the MSC. This instantaneous goal was the endpoint specified by the sum of all submovements initiated so far. We did not train the measurement model and instead assumed that it was ideal, i.e., it was identical to the encoding model used to generate the neural firing rates in the “neural encoder.” This was done to focus the analyses on the relative effectiveness of different neuron types (e.g., goal, position, velocity, position + velocity) and NOT on distortions introduced by a non-ideal decoder.

Kalman Filters represent the estimated state as Gaussian probability distributions, with a mean value representing the most likely state, and a covariance value representing the uncertainty in that state estimate. The state vector included position, velocity, acceleration, and goal estimates. For each simulation, the Kalman Filter estimated state (the mean of the Gaussian probability distribution) was initialized with the known starting position, zero velocity, and zero acceleration. Because these were known, the Kalman Filter was initialized with low uncertainty in the initial estimates of these quantities, with covariances 0.001 cm for position, 0.001 cm/s for velocity, and 0.001 cm/s^2^ acceleration, in each dimension x, y, and z.

Unlike the starting position, the initial planned goal was unknown to the Kalman Filter. To reflect the high uncertainty in the initial goal estimate, the Kalman Filter covariance for goal was set to a high value of 1000 cm in each dimension, which is more than ten times the longest edge of the workspace (Figure 3). The probability distribution of the initial goal estimate was essentially flat over the workspace, indicating high uncertainty in this estimate. The initial estimated goal state (the mean of the distribution) was set to the average of the endpoints of the initial submovements of the same set of experimentally recorded reaches used to train the trajectory model. However, because of the high covariance, the precise value was inconsequential. The blue traces in Figure 2 correspond to the decoded position, velocity, acceleration, and goal. The blue traces start at *t* = 0 with the initialized values but drift apart from the commanded trajectory due to the spiking noise added to the neurons, the numbers of neurons used, and imperfections in the trained trajectory model.

### Targets

The set of movements that we simulated represented outward-from-armrest target-oriented reaching, the kind of reaches that would be made by individuals from sitting positions. These are also the types of movements that we ultimately hope to restore to these individuals in a rehabilitation setting (Cornwell et al., 2012). For each of simulation parameters, 33 different reaches were simulated (Figure 3). The same targets were used for all parameter sets. The mean distance-to-target was 48.2 cm, the minimum distance was 27.98 cm, and the maximum distance was 68.69 cm.

### Performance metrics

We developed a performance metric that reflects the practical need for a BCI to both attain a target AND stably maintain the target for a functionally meaningful period. This metric is termed Minimum Attainable Target Radius (MATR), defined as the smallest target radius to which the decoded position moved and remained within for 1 s:

(7) $$\begin{array}{l} MATR=\underset{r}{\arg\min}\{\text{distance}\left(t+d\right)<r,\\ \;\forall \left|d\right|<\frac{1}{2},\frac{1}{2}<t<\left(t_{stop}-\frac{1}{2}\right)\} \end{array}$$

In this equation, the distance is the instantaneous distance to target. The variables *t* and *d* represent time, together indicating a sliding window of width 1 s. The MATR is the minimum *r* such that the distance is less than *r* for every time step inside the 1 s window. The variable *t*_*stop*_ represents the stop time. The first and last half-second of simulation were not included. An example of this is shown in Figure 2E. The gray rectangle represents the MATR. Note that target radius was not varied during the simulations—all of the reported MATR calculations were performed *post-hoc* on simulated trajectories. To characterize the smallest target radius achievable at various points in the workspace, we averaged the MATR over all 33 simulated targets (Minimum Attainable Target Radius over Targets, MATR-T). For a 3D view of sample simulated (decoded) trajectories with corresponding MATR for each target, see Figure 3.

Finally, each simulation to each target was repeated with 30 different sets of neuron parameters (preferred directions, tuning depths, baselines, etc.). To summarize MATR-T over neuron parameter sets, we took an additional mean over neuron parameters (MATR-T over Parameters, or MATR-TP) and also calculated the 95% confidence intervals of the mean. This confidence interval characterized the variation in MATR-TP due to different neuron parameter sets.

### Analyses performed

As mentioned above, two analyses were performed. The first analysis constrained the neuron tuning to represent the types of neurons that would be recorded in a practical BCI, with electrode arrays implanted in M1 (neurons tuned for position, velocity, or both) and in a goal-tuned region. The numbers of M1 and goal-tuned neurons were each varied from 0 to 50. We simulated Gaussian and linear goal neurons separately. The simulator stopped each movement at 3 s or 30 submovements, whichever came first, and ran at a 30 ms step size. The simulation shown in Figure 2 was performed under these conditions.

In the second analysis, we separated the movement time, the number of neurons, and the controller, from the performance of the three neuron types. We simulated 50 position, 50 velocity, and 50 goal neurons when movement time was not a limiting factor (we allowed 30 s of movement time or 300 MSC submovements). To understand the unique performance contributions of position and velocity neurons, cells that were simultaneously tuned for both position and velocity were not included. To investigate the influence of the MSC on simulated performance, we performed a number of movement simulations with the MSC included and then performed the same simulations with constant position and goal commands set exactly at the target location. Note that constant commanded velocities cannot reach the target and were not included in these simulations. In theory, a closed-loop controller such as the MSC should outperform the constant command signal and correct for any steady-state errors that may occur.

## Results

### Analysis 1: M1 position-velocity ensemble vs. goal ensemble

**Figure 4:**
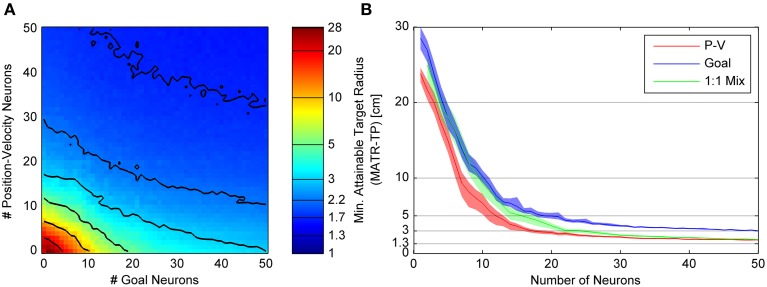
**Minimum attainable target radius for combinations of 20cm tuning width Gaussian goal and linear position-velocity neurons**. **(A)** shows the relationship between the minimum attainable target radius and the number of goal and position-velocity neurons. The colors represent the minimum attainable target radius averaged over 33 targets and again over 30 neuron sets (MATR-TP, see text). The contour line values are indicated by horizontal lines on the colormap bar. **(B)** shows three slices through the left panel. The blue trace (slice along the horizontal axis) represents the target radius vs. number of goal-only neurons. The red trace (slice along the vertical axis) represents the same for position-velocity neurons. The green trace (slice along the diagonal line through the plot) represents the same for a 1:1 mixture of goal and position-velocity cells, the diagonal of the left panel. The 95% confidence intervals are also shown.

**Figure 5:**
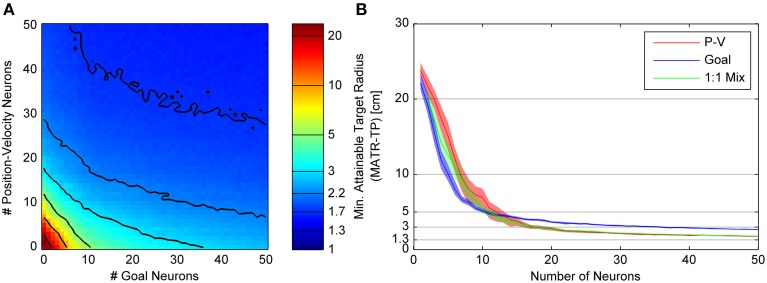
**Minimum attainable target radius for linear goal and linear position-velocity neurons**. This figure follows the same conventions as Figure 4, showing the relationship between minimum attainable target radius (MATR-TP, see text) and number of goal or position-velocity neurons on the left **(A)**. The right **(B)** shows the mean and 95% confidence intervals for MATR-TP, for goal-only neurons, position-only neurons, and a 1:1 mixture of these neuron types.

The relationships between the number of neurons and MATR are presented separately for Gaussian (Figure 4) and linear (Figure 5) goal tuning.

#### Gaussian goal tuning

Figure 4A illustrates the minimum attainable target radius (MATR-TP) (whose magnitude is represented by the colormap) for simulations run with different combinations of “goal neurons” and “position-velocity neurons.” Gaussian **goal** neuron ensembles of size 0 through 50 (represented on the horizontal axis) and **position-velocity** neural ensembles of size 0 through 50 (represented on the vertical axis) were simulated. Each simulation represented a 3 s reaching movement and (in this example) the Gaussian tuned goal neurons had an initial 20 cm standard deviation. The colors represent the MATR-TP for the corresponding number of goal vs. position-velocity neurons, with blue representing better performance and red representing poorer performance. The contour lines in Figure 4A correspond to the target radii indicated by the horizontal lines on the color bar of Figure 4A. The same reach targets and the same neuron sets were used for each combination of goal and position-velocity neuron number. These simulations indicated that increases in the number of both goal neurons and position-velocity neurons contributed to increased performance (i.e., decreases in MATR-TP) for target radii greater than 3 cm. However, MATR-TP decreased more quickly for increased numbers of position-velocity neurons than for increased numbers of goal neurons.

Figure 4B shows the MATR-TP (vertical axis) vs. the number of neurons (horizontal axis) for simulations performed with only position-velocity-tuned cells (red trace), with only goal-tuned cells (blue trace), and with a 1:1 ratio of goal and position-velocity cells (green trace). For each color, the middle lines represent the MATR-TP, and correspond to the information along three slices through Figure 4A (the vertical axis, horizontal axis, and the diagonal). The 95% confidence intervals of the MATR-TP are also shown, representing the variation in MATR-TP caused by repeating the same simulations with different neuron parameter values. Non-overlapping confidence intervals between the various curves indicate statistically significant differences. For small numbers of neurons (<5) and large targets (>15 cm) the position-velocity and the 1:1 mixture confidence intervals overlap, indicating similar performance. For small numbers of goal neurons (<15) and targets >5 cm the goal and 1:1 mixture confidence intervals overlap, also indicating similar performance. For the same number of neurons, though, goal neurons perform worse than position-velocity neurons. Note that position-velocity neurons reached a MATR-TP asymptote of ~2 cm using approximately 30 neurons. The 1:1 mixture reached a similar asymptote at approximately 30 neurons, while the goal neuron asymptote was approximately 1.3 cm higher. The green curve was generally located between the red and blue curves, indicating that the mixture of neurons did not perform better than an equivalent number of position-velocity neurons. However, the addition of goal neurons to any particular number of position-velocity neurons did improve performance. For instance, for 10 position-velocity neurons (red trace) the MATR-TP was ~7 cm. In comparison, 20 mixture neurons (green trace) contains 10 position-velocity and 10 goal neurons, and the MATR-TP was ~3.5 cm. However, note that the MATR-TP for 20 position-velocity (red trace) neurons was ~2.8 cm. Therefore, the MATR-TP was reduced less by 10 additional goal neurons than by 10 additional position-velocity neurons. A Kruskal-Wallis test comparing the three curves indicated that the differences were statistically significant (*p* < 0.001). Pairwise comparisons between the curves were also significant (*p* < 0.005).

The corresponding figures for different Gaussian goal tuning widths (standard deviations) are included in the Supplemental Materials. Note that the goal neuron MATR-TP was smaller at 20 and 30 cm tuning widths, and larger at 10 and 40 cm tuning widths. Also, note that for 10 cm Gaussian standard deviation, the contour lines that originate at above 10 position-velocity neurons have positive slopes in the x-y plane, indicating that MATR-TP actually increased with additional neurons, before leveling off and decreasing with higher numbers of neurons. For 40 cm Gaussian standard deviation, MATR-TP decreased with additional goal neurons except for the transition from 1 to 2 goal neurons. For the other goal tuning widths, MATR-TP decreased with additional goal neurons.

#### Linear goal tuning

Figure 5 shows the MATR-TP for simulations run with *linear* goal neuron populations of size 0 through 50 and position-velocity neural populations of size 0 through 50. Other than the different type of goal tuning, the panels and labeling are the same as in Figure 5. Compared to Figure 4A, the contour lines close to the origin in Figure 5A have slope ≤1, indicating that adding goal neurons would have larger performance benefit than adding P-V neurons initially. However, for greater than 10 neurons the contour spacing along the horizontal axis increases relative to the vertical axis spacing, indicating that additional P-V neurons have a more beneficial impact on performance than adding goal neurons. This effect is also visible in Figure 5B, where the MATR-TP confidence intervals for goal are less than for position-velocity neurons at less than 10 cm, and overlap at up to 15 neurons, and do not overlap for larger numbers of neurons where the P-V curve is consistently lower (i.e., better performance). The 1:1 mixture of P-V and goal neurons never performed better than position-velocity neurons only or goal neurons only. Position-velocity and 1:1 neuron populations reach asymptotic performance of ~2 cm in approximately 30 neurons, and goal neuron performance asymptote was approximately 1.3 cm higher. As in Figure 4B, adding goal neurons did improve upon P-V only performance, but the improvement was not as great as it was when additional P-V neurons were included. A Kruskal-Wallis test comparing the three curves indicated that the differences were statistically significant (*p* < 0.001). Pairwise comparisons between Goal and PV (*p* = 0.78), 1:1 and PV (*p* = 0.24), and 1:1 and Goal (*p* = 0.091) were not significant.

### Analysis 2: 50 position, velocity, or goal neurons, with longer reach durations

**Figure 6:**
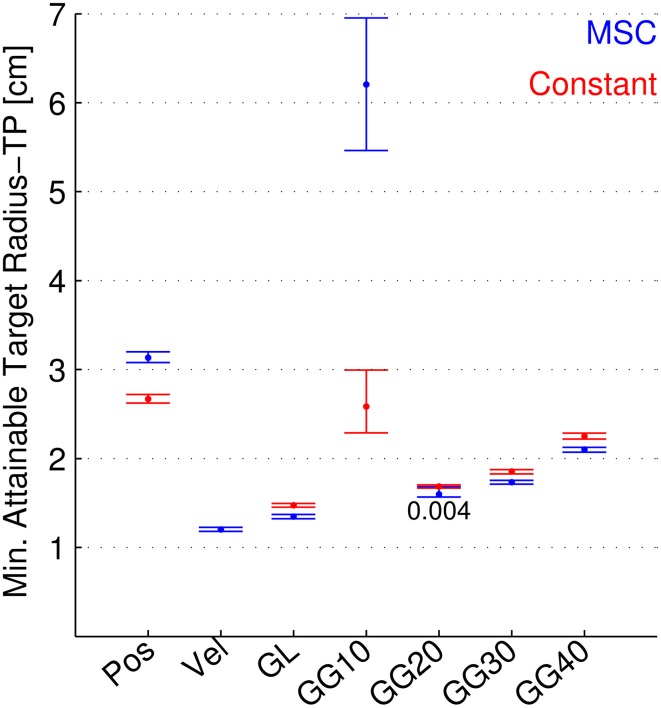
**Minimum attainable target radius for separate neuron types, both continuously modulated and constant**. The minimum attainable target radius (MATR-TP, see text) is shown for position, velocity, linear goal (GL), Gaussian 10 cm tuning width goal (GG10), 20 cm (GG20), 30 cm (GG30), and 40 cm (GG40). Blue error bars indicate mean and 95% confidence intervals for the MATR-TP using 50 of the respective neurons and the Multiple Submovement Controller. Red error bars indicate the same metric for constant (i.e., not corrected) trajectory commands, for position and goal tuning.

**Figure 7:**
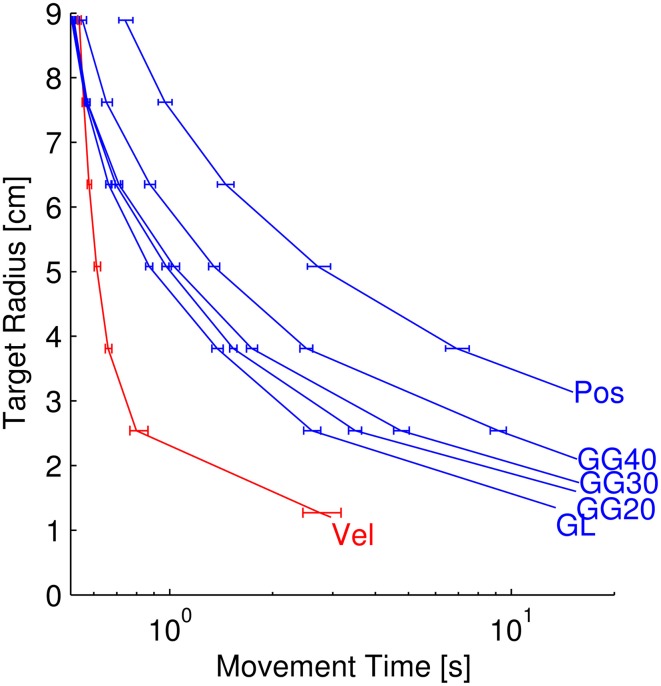
**Target radius vs. movement time for separate neuron types**. Each curve represents the relationship between target radius and movement time for simulated reaches using 50 position, velocity, or goal neurons of the respective types (GL, linear goal; GG, Gaussian Goal of 20, 30, or 40 cm standard deviation). The bottom-right-most points of each series represent the minimum achievable target radius (MATR-TP) from Figure 6. The error bars represent the 95% confidence interval of the movement times at particular target radii.

**Figure 8:**
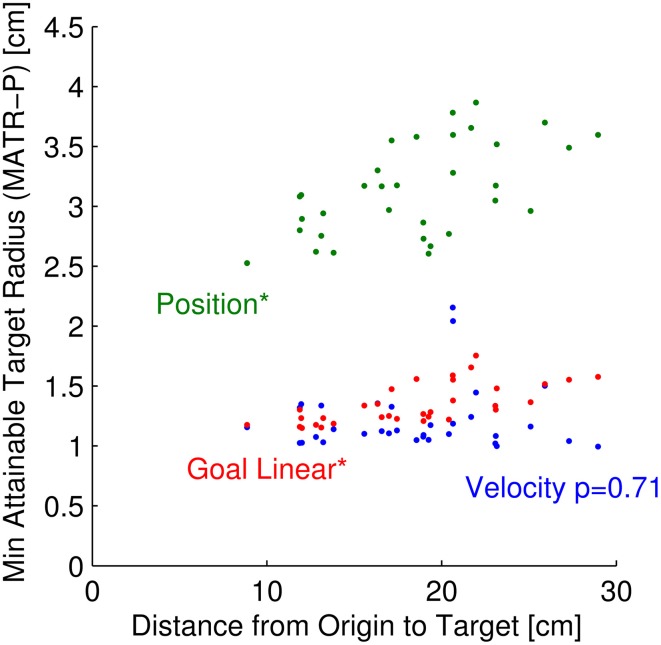
**Minimum attainable target size vs. distance from origin to target**. For each of 33 targets, the relationship between minimum attainable target radius, averaged across neuron parameter sets (MATR-P) is plotted against the distance from origin to target. The green dots represent this relationship for 50 position neurons. The blue and red points represent this relationship for 50 velocity and 50 linear goal neurons, respectively. The relationship between MATR-P and distance from target to origin is statistically significant for position and linear goal neurons (*p* < 0.001, labeled above as ^*^), but is not significant for velocity neurons (*p* = 0.71).

For the second analysis, the MATR for each neuron type, the relationship between target radius and movement time, and the relationship between target radius and distance from origin were explored, and the results are presented in Figures 6–8, respectively. Specifically, movement times up to 30 s and up to 300 submovements (for the MSC simulations) were allowed.

#### Minimum attainable target radius

Figure 6 shows the minimum attainable target radius (MATR-TP) (vertical axis) plotted for different neuron types (horizontal axis), with 50 neurons for each type. The neuron types included position-only neurons (labeled Pos), velocity-only neurons (labeled Vel), goal-only neurons with linear tuning (GL), and goal-only neurons with Gaussian tuning at 10, 20, 30, and 40 cm tuning widths (standard deviations, labeled GG10, GG20, GG30, and GG40). The blue error bars represent performance using the MSC. The means and 95% confidence intervals depicted by the bars represent the MATR-TP and the variation in MATR-TP due to neuron parameter sets. The red error bars indicate the corresponding values when the command signals for position and goal were constant, set to the target position (the start position was not changed). Non-overlapping confidence intervals indicate statistically significant differences. For the case where intervals overlapped, the *p*-value is shown. Constant commanded velocities cannot reach the target and were not included in this figure.

Using the MSC, velocity tuned cells consistently provided the smallest attainable targets. Also, linear and Gaussian goal-tuned cells (except for 10 cm standard deviations) performed better than position-tuned cells. Gaussian goal tuned neurons with 10 cm standard deviation performed poorest among all tuning types. Note that the MATR-TP of goal-tuned cells in this figure (with up to 30 s of movement time allowed) were smaller than in Figures 4, 5 (where movement time was limited to 3 s). This indicates that short movement times limit the minimum attainable target radii for goal-tuned neurons.

The MSC performed better than the constant controller for linear, 20 (*p* = 0.005), 30, and 40 cm goal tuning. For position and 10 cm goal tuning, the MSC was worse than the constant controller.

#### Target radius vs. movement time

The amount of time required to achieve targets of a particular radius decreased as the target radii increased. We investigated this relationship *post-hoc* with MSC-controlled reaches (Figure 7). This figure contains six data series corresponding to the neuron types from Figure 6, with the exception that the poorest-performing 10 cm standard deviation Gaussian tuning condition is not included. Each curve represents the target radius vs. movement time relationship for the labeled neuron type. The error bars indicate the 95% confidence intervals of the movement times at specific radii. Like the MATR-TP, the movement times were calculated as means of means over targets and neuron parameter sets. The lower right point of each data series represents the MATR-TP and the corresponding movement time when that target radius was attained. The y-values of these points are identical to the error bar means from Figure 6. The other points along the curves represent the movement times required to reach larger targets. Note that the horizontal axis has a log scale.

Velocity neurons enabled the smallest target as well as the shortest movement times, and position neurons allowed larger targets and required longer movement times for a given target radius. Goal neurons enabled performance levels between those of position and velocity neurons. For goal and position-tuned neurons, the MATR-TP was attained at greater than 10 s of movement time. Note that as the target radius increased (top left of Figure 7), the difference between the movement times decreased. The velocity and goal curves began to overlap at 7.62 cm target radius. At this target radius the differences in movement times between velocity and linear goal (*p* = 0.361), velocity and 20 cm goal tuning (*p* = 0.036), velocity and 30 cm goal tuning (*p* = 0.042), were not significant. The other comparisons (velocity to position, and velocity to 40 cm goal tuning) were significant (*p* < 0.001). By 8.89 cm target radius, the mean velocity movement times were larger than the goal movement times. The velocity and 40 cm goal tuning traces had overlapping confidence intervals and the difference was not statistically significant (*p* = 0.121).

#### Target radius vs. distance of target from origin

The MATR was related to the distance from target to the origin (Figure 8) for some neuron modalities but not others. For this figure, the MATR was averaged across neuron parameter sets only (MATR-P), and is plotted for each of the 33 targets as a function of the distance from origin to the target. The green points represent the MATR-P for 50 position cells, the red points represent the same for linear tuned goal cells, and the blue points represent the same for velocity cells. Note that the velocity MATR-P was not a function of distance from origin to target (*p* = 0.71). However, the MATR-P for linear goal and position tuned cells increased with increasing distance from origin to target (*p* < 0.001).

## Discussion

We have developed a model of a closed-loop BCI for controlling human arm movements that uses a model of point-to-point arm movement trajectories (including error correction), constructs an ensemble of movement-related cortical neurons that encode these ideal movements using realistic firing characteristics and noise properties based on literature reports, and then decodes the movement commands that would be expected from a practical decoder. We performed a number of simulations to explore the likely contributions of different combinations of position, velocity, and goal-tuned neurons, as well as the impact of the number of neurons, the tuning properties of the goal-based neurons, the allowed movement times, and the distance from origin to target. These simulations suggest that these neuron types all contributed to improve performance (i.e., decrease the size of reachable targets).

### Relative utility of goal neurons

Most previous studies that evaluated goal information content in neural signals have used a classification approach to predict which of a pre-defined set of targets the participant intends to reach (Santhanam et al., 2006; Achtman et al., 2007; Shanechi et al., 2013a; Aflalo et al., 2015). Our approach uses the MATR metric to characterize how well targets that are arbitrarily placed in the workspace can be reached with a given number of neurons. This allows goal neuron performance to be directly compared to that of position or velocity neurons.

In most cases, performance improved and eventually saturated as the number of neurons increased (Figures 4, 5 and Supplementary Figures S2, S3). For neurons tuned for position and/or velocity, the performance saturated at ~30 neurons, which would allow for targets of ~2 cm to be reached within 2 s (not including the dwell time). Goal neuron performance also saturated at ~30 neurons, allowing for targets of 3–5 cm to be reached in 2 s (Figures 4, 5 and Supplementary Figures S2, S3). While goal neurons did not allow for as much precision as a similar number of M1 neurons tuned for both position and velocity, the range of performance provided by goal and position-velocity tuned neurons is likely fast and accurate enough for many practical arm reaching tasks. In addition, given >5 s of movement (not including the dwell time), 50 goal neurons were able to specify targets of 2–3.5 cm (Figure 7). One exception was the 10 cm tuning width goal neurons (Figure S1). This case will be discussed in the next section.

Our results suggest that given a limited number of recording electrodes, maximizing the number of M1 electrodes (neurons tuned for position and/or velocity) would be more beneficial than recording neurons from a goal-tuned cortical region, with a few caveats. Our study did not account for movement-related neural activity in different brain areas that occur at different times (Shibasaki and Hallett, 2006). Though it takes more time for a goal neuron ensemble to specify targets as small as the same number of velocity neurons can (Figure 7), the goal-related neurons may modulate earlier in movement planning than position or velocity neurons. The effect of this “early information” should be investigated in a future study.

Even if goal neurons could only specify larger targets, there may be advantages to their use. Goal information can be used to constrain (Srinivasan et al., 2006; Yu et al., 2007; Corbett et al., 2012; Shanechi et al., 2013b) the decoded trajectory, potentially reducing the cognitive burden associated with specifying that trajectory. To our knowledge, the effect of goal-tuned neurons on cognitive burden has not yet been tested in humans. This would be important to study in a future human BCI participant.

Because performance saturates with increasing numbers of neurons, our study suggests that small neuron ensembles of <50 neurons can provide most of the performance that a much larger neuron ensemble would provide. Also, at greater than 20–30 neurons the confidence intervals for the MATR-TP are very narrow, indicating that in the saturation region there is minimal variability in performance due to different sets of neuron parameters. This requires that the tuning functions of each neuron be well characterized. This could be accomplished via adaptive closed-loop decoding, where neurons adapt to the BCI over time and thus become better tuned for movement-related parameters over the course of decoder training (Carmena, 2013).

### Effect of Gaussian goal tuning width on performance

The effect of the Gaussian goal tuning width (standard deviation) on performance is shown in Figure 6. The ability of Gaussian-tuned neurons to represent goal location depend on how much the firing rate changes, on average, over the entire workspace (Zhang et al., 1998). If the tuning widths were too narrow (Brown and Bäcker, 2006) as in the 10 cm case (Figure S1), the firing rates over most of the workspace would be in the tails of the Gaussian distribution. A similar phenomenon would occur if the tuning widths were too wide. The average change in firing rate per change in goal position would be low, and the decoder's ability to detect changes in commanded goal position would be diminished. Performance for 10 cm tuning widths (Figure S1) at 5–10 goal neurons improved at above 20–30 goal neurons as the workspace was “covered” by a sufficient number of narrowly tuned goal neurons. For the workspace size simulated, the 20 cm tuning width provided the best performance. However, our results suggest that goal neurons with a wide range of tuning widths can contribute to improve decoding performance.

At less than three goal neurons at 10 and 40 cm tuning width, performance initially worsened before improving with additional neurons (Figures S1, S3). This reflects the relatively poor information represented by small numbers of 10 or 40 cm tuning width neurons, combined with the simulation stop parameters. With very few neurons the decoded trajectories poorly matched those expected by the MSC, which initiated 30 submovements quickly. The simulations stopped before the decoded trajectory drifted too far from the start position. With a few more neurons (e.g., 2 or 3 goal neurons in Figure S1), the MSC corrections occurred less quickly but control was still poor, and the decoded trajectories drifted away from the target resulting in larger MATR. With larger numbers of goal neurons the decoded trajectories approached the target, reducing the MATR.

### Effect of distance-from-origin-to-target on performance: Signal to noise ratio

The relationship between MATR and distance from origin to target (Figure 8) suggests an explanation for the performance contributions of the different neuron types. Near the target, the commanded velocities were near zero, so the firing rates were near their baselines. The commanded positions and linear goal tuning were not near zero—they were at baseline plus an offset that was proportional to the distance from the origin. However, the magnitudes of the small corrections near the targets were not proportional to this distance. In addition, large magnitude offsets drove the firing rates closer to saturation. Therefore, the SNR for position and linear goal neurons decreased with increasing distance from origin, while the velocity neuron SNR remained constant. These phenomena are reflected in the minimum attainable target size (Figure 8). Thus, for the position and goal neurons simulated, the reference frame of the neurons and the BCI task workspace both affected performance.

Careful characterization of neuron reference frames may allow BCI tasks to be designed such that the SNR of the recorded neurons is optimized. Understanding neuron reference frames is an ongoing effort (Sakata et al., 1980; Kettner et al., 1988; Schwartz et al., 1988; Alexander and Crutcher, 1990; Galletti et al., 1993; Shen and Alexander, 1997; Moran and Schwartz, 1999; Genovesio and Ferraina, 2004; Saito et al., 2005; Churchland et al., 2006; Pesaran et al., 2006; Aflalo and Graziano, 2007; Wang et al., 2007; Bhattacharyya et al., 2009; Hadjidimitrakis et al., 2011) and is difficult because neurons do not have to be tuned for any particular coordinate system and are even modulated by non-movement-related parameters such as expectation of reward (Musallam et al., 2004). Again, adaptive closed-loop decoding (Carmena, 2013) may reduce the need to fully characterize the original reference frames of the neurons as they learn to control the BCI.

### Multiple submovement controller

The MSC (Liao and Kirsch, 2014) is based on the hypothesis that target-oriented arm reaching movements consist of a series of overlapping submovements (Lee et al., 1997; Burdet and Milner, 1998; Rohrer et al., 2004; Fishbach et al., 2007) that each represent corrections to the movement based on feedback AND whose summation represents the commanded trajectory. Under this hypothesis, the early portion of each reach is not specified precisely (Rand and Shimansky, 2013) and tends to undershoot the target (Worringham, 1991), necessitating corrections in order to achieve the target. Thus, the intended goal does not necessarily coincide with the target early in the reaching movement (Lyons et al., 2006). We chose the MSC to drive the simulation of neural data because it models the commanded goal trajectory in addition to the position and velocity trajectories. A model of the goal trajectory is required for simulation of goal-tuned neurons.

The MSC was able to guide the decoded position to the vicinity of the target under various tuning conditions and numbers of neurons (Figures 4–6). However, the MSC was not perfect. Ideally, it should achieve smaller targets than a constant command signal would. The MSC training process may have introduced imperfections—larger datasets, datasets with more precise experimentally recorded reaches, different data preprocessing, or a different ANN architecture may have improved the MSC's performance.

We made the simplification to use the same MSC for all cases, although it is possible that actual BCI users adopt different control strategies depending on the different numbers or types of neurons. The possible effect of our simplification would be more pronounced with small numbers of neurons, or, when the neurons each represent less information as in the 10 cm Gaussian case. This could be addressed by using a controller that models the optimization process that occurs as users learn to operate the BCI, but this is beyond the scope of the current study.

Optimal control theory may provide a solution for this problem. One group developed a velocity feedback controller that drives a set of simulated velocity neurons, pushing the decoded position to a target (Kowalski et al., 2013; Lagang and Srinivasan, 2013). It may be possible to extended this controller to also command a time-varying goal signal to optimally drive the decoded position to the target. Eventually, dynamics of the decoded signal (when coupled to a robotic or FES-enabled arm) could also potentially be incorporated into the controller.

Other alternatives include using human participants in the loop whose real-time arm kinematics drive simulated neural spiking (Cunningham et al., 2010). This approach makes fewer assumptions about the control strategy but is difficult to scale to hundreds or thousands of simulations. Another group assumed that the mapping between commanded and decoded movement directions could be learned, so the commanded trajectories always resulted in straight movements to the targets (Chase et al., 2009), even though the study was essentially open-loop. It is yet unclear how a time varying goal signal should be added to these approaches, but they are worth exploring further.

### Suitability of Kalman Filter approach to goal signal integration

Our simulations confirm that the Kalman Filter with a linear trajectory model augmented with goal (Mulliken et al., 2008a; Corbett et al., 2012) is capable of utilizing that goal information to improve the decoded trajectory, as the addition of goal neurons improved performance (Figures 4, 5) in every case except for 10 cm standard deviation Gaussian tuning (Figure S1). However, more sophisticated methods for incorporating goal have been proposed (Srinivasan et al., 2006; Srinivasan and Brown, 2007; Corbett et al., 2012; Lawhern et al., 2012; Shanechi et al., 2013a). Our closed-loop simulation approach allows these methods to be directly compared and would be a useful future study.

### Impact of assumptions and comparisons with other studies

This study made the assumption that the decoder had knowledge of the ideal neuron tuning functions. This was done explicitly to eliminate any confounds that could result from the training of a practical decoder, thus allowing us to compare the utility of position, velocity, and goal signals for generating appropriate arm trajectories *per se*. It is possible, however, that practical decoders trained on experimental or simulated neural data will perform somewhat differently and increase or decrease the relative performance contributions of neuron types. A future simulation study could thus use a decoder that is trained on simulated neural data, and the analyses could then be repeated using that non-ideal decoder.

This assumption makes it difficult to directly compare our simulation results to those reported in experimental BCI studies. Also, differences between tasks, success conditions, and performance metrics further complicate direct comparisons. Collinger et al. (2013) reported that a human participant could, with ~50 velocity neurons, hit ~90% of targets at 8 cm radius. Hochberg et al. (2012) reported that a human participant could hit ~95% of targets where the target radius was approximately 13 cm (3 cm target radius plus 10 cm of robot hand aperture). The number of neurons used was not reported. In comparison, the MATR in our simulation are less than 3 cm at greater than 30 position-velocity neurons. It would be interesting to explore how this would change using a decoder trained on simulated neural data rather than an ideal decoder.

In terms of performance vs. number of neurons, Aflalo et al. showed that hit rate saturated at ~90% using a decoder trained on 20–30 experimentally recorded neurons, when neurons were selected using a greedy algorithm in order of contribution to task performance. However, using random instead of greedy neuron selection, it took ~100 neurons to reach ~90% hit rate (Aflalo et al., 2015). In our simulation we used a random neuron selection and an ideal decoder, and performance saturated at 20–30 neurons. We expect the performance to saturate at a larger number of neurons if we used trained decoder rather than an ideal decoder, but further simulations are required to determine the saturation curve.

We also made assumptions regarding goal neuron tuning depths. The relationship between goal neuron tuning depths and their relative contribution to the size of reachable targets was not investigated. However, our assumptions about tuning depths likely over-estimate the goal neuron contributions. For instance, linear goal neurons represent the same amount of workspace as position-tuned neurons, but the linear goal neurons were more deeply tuned. Even with this potential over-estimation, the goal neurons did not contribute as strongly as velocity-tuned neurons. This issue is addressable using the closed-loop BCI simulator and would be an interesting future study.

## Conclusion

Goal, position, and velocity-tuned neurons all strongly contributed to decrease the size of targets that could be reached by a simulated BCI. However, goal neurons did not contribute as strongly as velocity neurons did. Therefore, for BCIs that require high precision, additional recording channels in M1 would be more useful than additional channels in brain areas that contain goal-tuned neurons. However, although this study does not address it, there may be other benefits to using goal neurons such as decreased cognitive burden that would still make them useful for a practical BCI.

This study also contributed a BCI simulator capable of making error corrections in closed-loop, based on a model of human movement previously developed in our lab. We anticipate that approaches incorporating similar, or more advanced, models of error corrections will be useful development tools for future BCI algorithms, especially those that incorporate goal-tuned neurons, and allow them to be tested in simulated closed-loop prior to *in vivo* experimental study.

### Conflict of interest statement

The Associate Editor Florentin Wörgötter declares that, despite being a member of the PhD defense committee of reviewer Jan-Matthias Braun, the review process was handled objectively. The authors declare that the research was conducted in the absence of any commercial or financial relationships that could be construed as a potential conflict of interest.
