# Supplementary material for: Velocity neurons improve performance more than goal or position neurons do in a simulated closed-loop BCI arm-reaching task
Source: Front Comput Neurosci. 2015 Jul 14;9:84. doi: 10.3389/fncom.2015.00084 (PMC4500927; doi:10.3389/fncom.2015.00084)
Supplement: Supplementary file 1 [file TextandFigures.DOCX]

***Supplementary Material***

Velocity neurons improve performance more than goal or position neurons do in a simulated closed-loop BCI arm-reaching task.

James Y. Liao^1,2^*, Robert F. Kirsch^1,2^

^1^Cleveland Functional Electrical Stimulation Center, Cleveland, Ohio 44106, USA

^2^Department of Biomedical Engineering, Case Western Reserve University, Cleveland, Ohio 44106, USA

***Correspondence:**

Dr. James Y. Liao

Medical Scientist Training Program

Case Western Reserve University

School of Medicine T401

10900 Euclid Ave.

Cleveland, OH 44106-4936, USA

james.liao@case.edu

1. **Supplementary Code**

We have provided sample code to allow users to explore the simulator. The functionality allows users to simulate reaches to all 33 targets using a single set of neuron parameters. Users can specify the number of velocity, position, or goal neurons to use (up to 200 of each type). Users can also specify the type of goal tuning: linear, 10cm standard deviation, 20cm standard deviation, 30cm, and 40cm. At this point, only one kind of goal tuning can be used at once.

The main file is called BCISimSample.m, this file loads the appropriate data files and Simulink model. The simulator is implemented in Simulink R2012b as a .mdl file and cannot be run directly, its workspace is set up via the various Matlab scripts. The code has been tested in Matlab R2012b.

The files are contained in zip file. To use, unzip them all to the same directory and run BCISimSample.m. Parameters can be adjusted by editing BCISimSample.m.

Sample trajectories to five targets made using 10 goal neurons, 1 position neuron, 5 velocity neurons, and 4 position-velocity neurons are shown in Supplementary Figure S4.

1. **Description of Ideal Behavior of Multiple Submovement Controller**

The Multiple Submovement Controller consists of three ANNs. The first ANN detects, based on kinematic features of the ongoing movement, when a correction should be made to the commanded trajectory, in order to bring the decoded trajectory toward the target. This ANN was trained on actual recorded reaching movements (see Liao and Kirsch 2014 for complete description).

Once it is determined that a correction is necessary, a second ANN predicts the amplitude of this submovement. As discussed in the Methods section, this ANN uses the start position, target position, decoded position, decoded velocity, decoded acceleration, and the predicted position at the end of the current submovements, to make this prediction (Liao and Kirsch 2014).

A third ANN predicts the duration of the new submovement. Together, the ANNs predicted the initiation time, the amplitude (in three dimensions), and the duration of the new submovement. These parameters are enough to specify a minimum-jerk submovement (see Equation 1).

This new submovement’s position, velocity, acceleration, and goal trajectories are linearly added to the corresponding trajectories for the previous submovements, forming the new commanded position, velocity, acceleration, and goal trajectories (see Equation 2).

Supplementary Figure S5 shows two sample simulations. The thick red traces represent the commanded position (Equation 2), which is the sum of the individual green submovement traces (each represented by Equation 1). The thin red traces represent the commanded goal (Equation 2). The commanded trajectories are limited to remain in the workspace, so the red traces rail at approximately -12 cm and -19cm in the X and Y dimensions, respectively. The blue traces represent the decoded position trajectory. The black traces represent the position of the target. Finally, the purple traces represent the predicted position at the end of the current submovements (PACSM).

$\begin{matrix} \mathrm{PACSM}\left(t\right)=pos_{fused}\left(t\right)+\mathrm{remainingDistance}(t) \\ \mathrm{remainingDistance}(t)=pos_{command}\left(t_{end}\right)-pos_{command}\left(t\right) \\ t_{end}=\max\left(t_{0,i}+t{}_{d,i}\right) \end{matrix}$ (Eqn S1)

The PACSM is the current fused position plus the remaining distance in the commanded position trajectory. The end of the commanded position trajectory occurs at time $t_{end}$, which is the maximum of the end times of all of the submovements so far.

The ideal behavior of the amplitude prediction ANN is to generate a new submovement whose amplitude brings the PACSM closer to the target. Submovements that begin when the PACSM has not yet reached the target should have amplitudes that bring the decoded position towards the target. In Supplemental Figure S5, if the purple PACSM trace is above the black target trace at the moment the submovement initiates, the submovements should be pointed down in the negative x, y, or z direction. If the purple PACSM trace is below the black target trace at submovement initiation, the submovements should be pointed up in the positive x, y, or z direction.

There are two instances in Supplementary Figure S5, top panel, where submovements appear to bring the fused trajectory back towards the starting position. In these cases, when the submovements begin, the PACSM has already gone past the target in the x dimension. That is, the current set of submovements is expected to take the decoded trajectory too far past the target in the x dimension. The predicted submovements therefore are pointed away from the target, in the x dimension. The direction of these submovements is the correct behavior for the MSC as currently designed.

Note that the reason the initial submovement of each reach does not go directly to the target is because the ANN was trained on experimentally recorded reaching. Submovements decomposed from experimentally recorded human reaching do not directly go to the target (Worringham 1991; Lyons et al. 2006).

1. **Supplementary Figures**


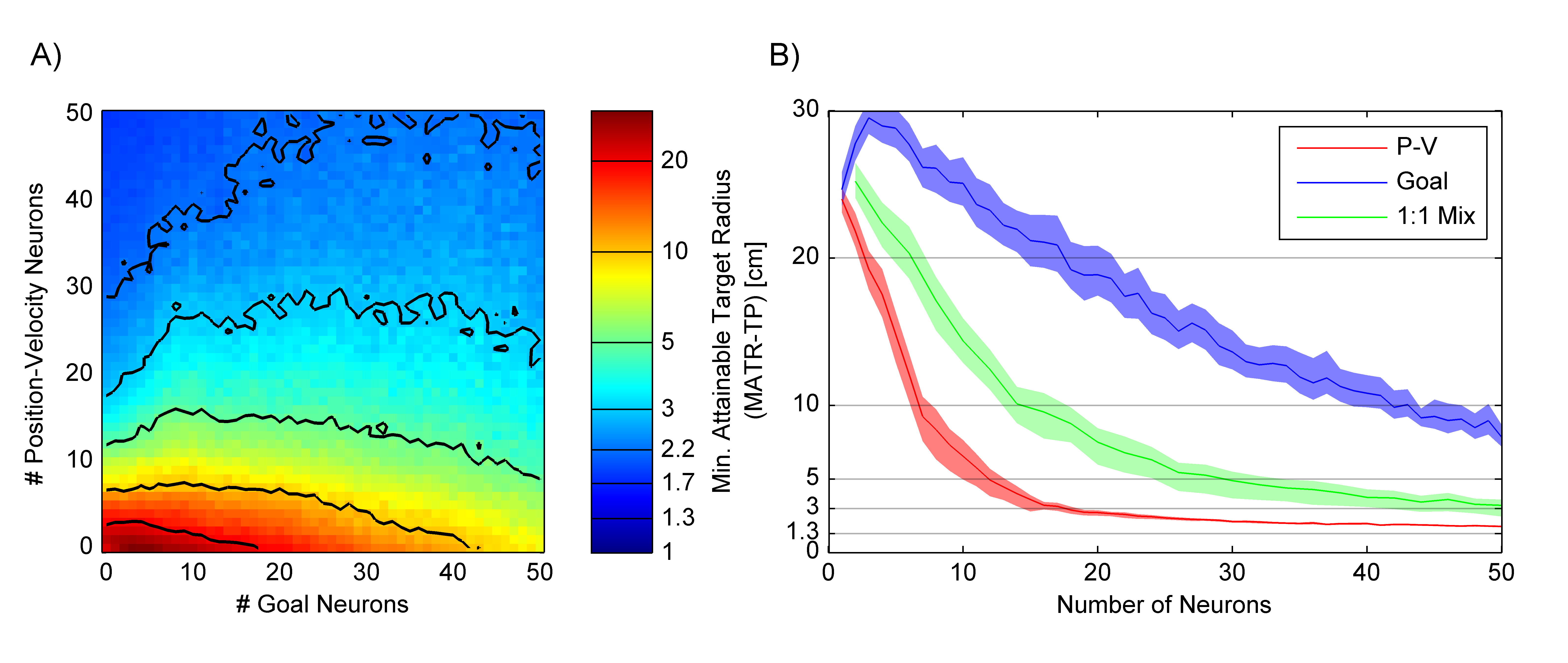


**Supplementary Figure S1: Optimal Target Size for 10cm Tuning Width Gaussian Goal and Linear Position-Velocity Neurons.** This figure follows the same conventions as Figures 3 and 4, showing the relationship between optimal target size and number of goal or position-velocity neurons on the left (a). The right (b) panel shows the mean and 95% confidence intervals for optimal target size, for goal-only neurons, position-only neurons, and a 1:1 mixture of these neuron types.


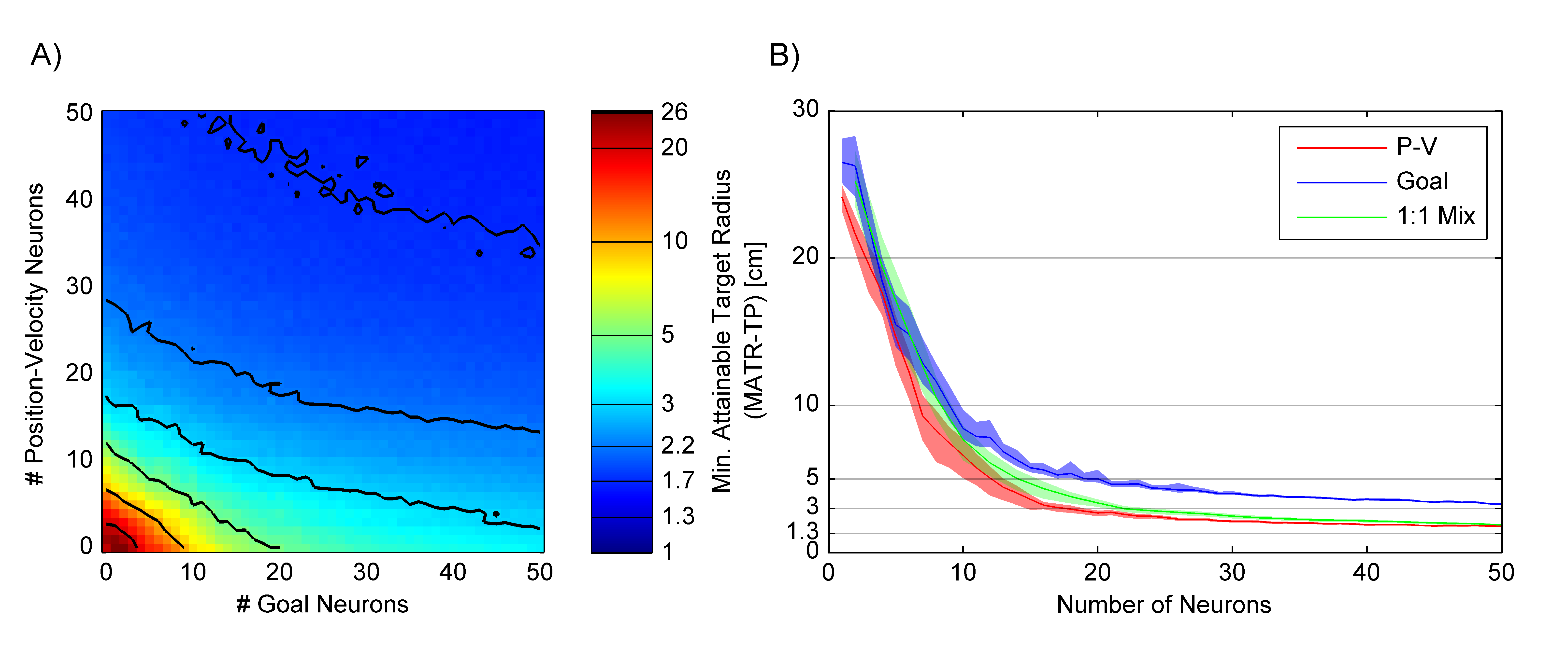


**Supplementary Figure S2: Optimal Target Size for 30cm Tuning Width Gaussian Goal and Linear Position-Velocity Neurons.** This figure follows the same conventions as Figure S1.

**
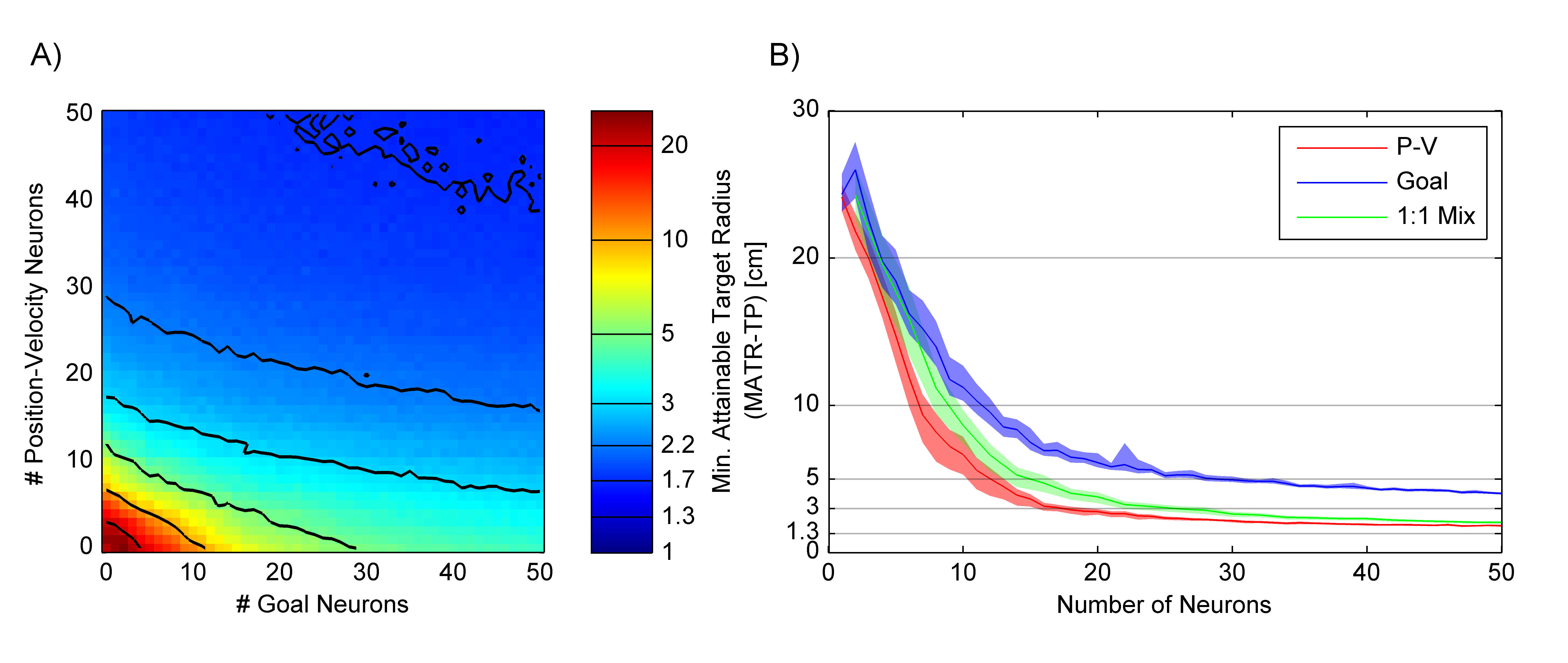
**

**Supplementary Figure S3: Optimal Target Size for 40cm Tuning Width Gaussian Goal and Linear Position-Velocity Neurons.** This figure follows the same conventions as Figure S1.


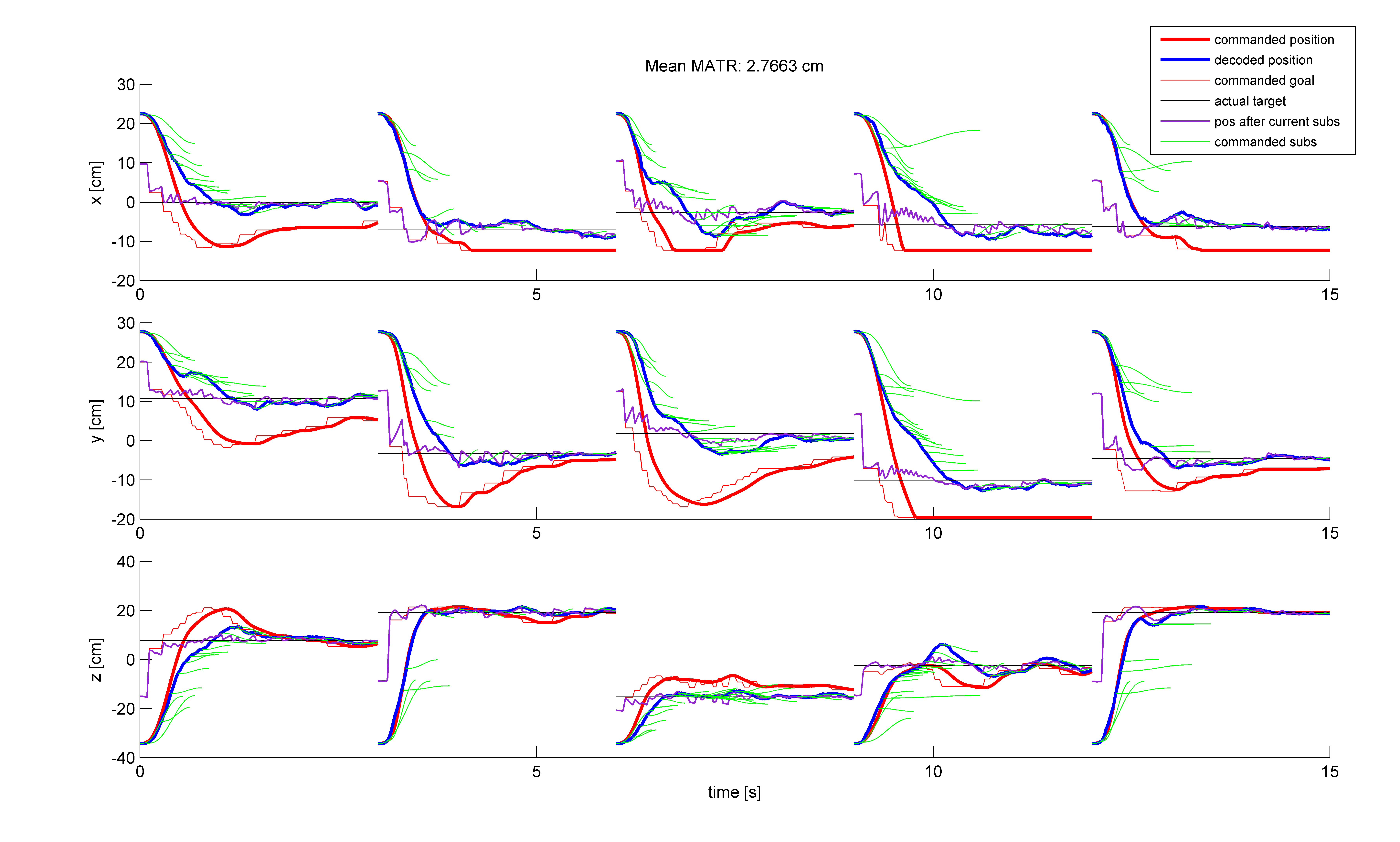


Supplementary Figure S4: Sample trajectories to five targets generated by the provided sample code. The three subplots correspond to x, y, and z position coordinates plotted against time. Five simulated reaches to 5 targets are represented. The reaches originate from the same starting position and the targets are indicated using thin black traces. The thick red traces represent the commanded position. The thick blue traces represent the decoded position. The thin red traces represent the commanded goal. The green traces represent the individual submovements commanded by the Multiple Submovement Controller (MSC). Finally, the purple traces represent the position after the current submovements predicted by the MSC. For these simulations, 10 goal neurons, 1 position neuron, 5 velocity neurons, and 4 position-velocity neurons were used. The mean MATR over these five reaches is 2.77cm.


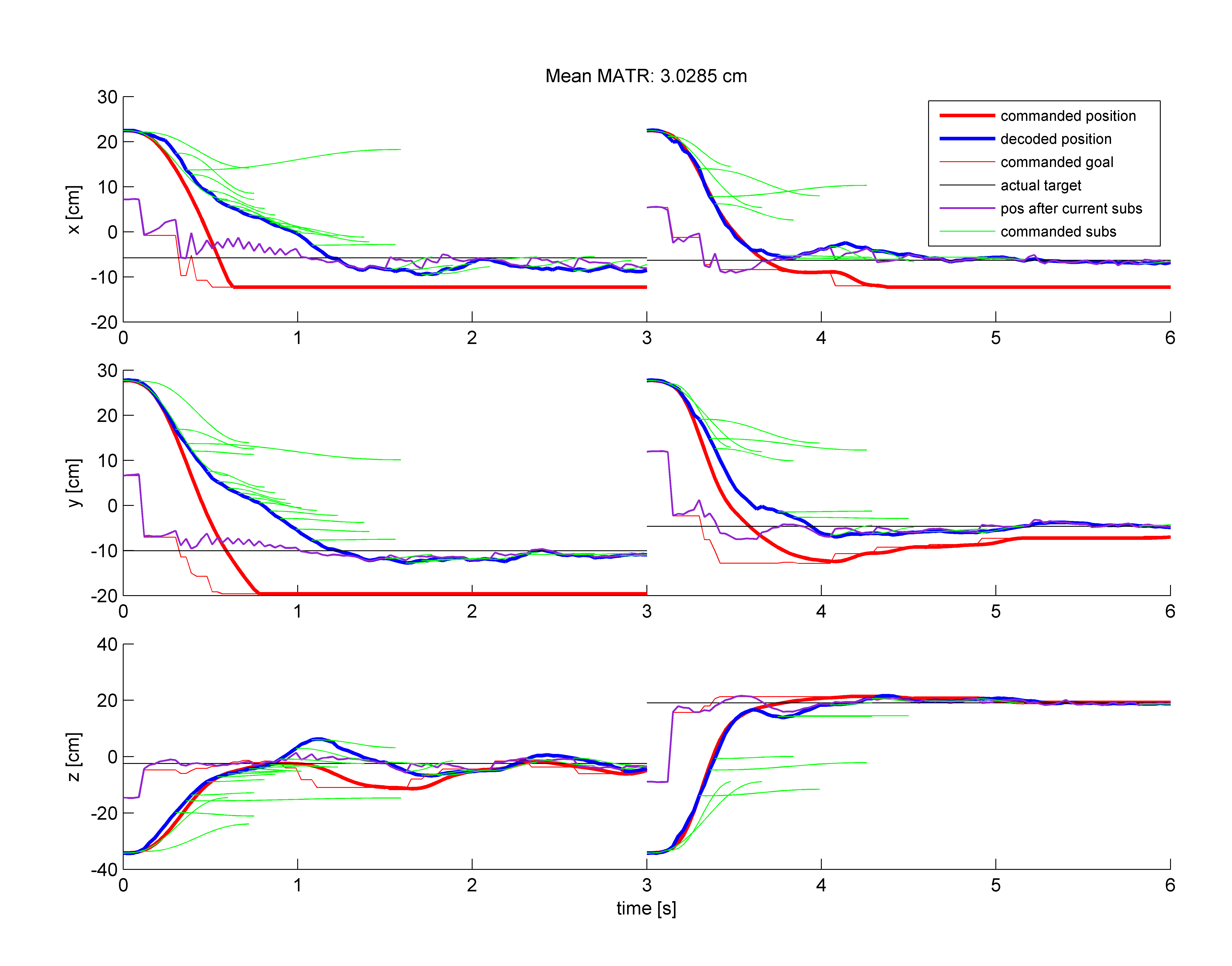


**Supplementary Figure S5: Enlarged version of columns 4 and 5 from Figure S4.** This figure follows the same conventions as Figure S4.
